# Local Membrane Curvature Pins and Guides Excitable Membrane Waves in Chemotactic and Macropinocytic Cells - Biomedical Insights From an Innovative Simple Model

**DOI:** 10.3389/fcell.2021.670943

**Published:** 2021-09-15

**Authors:** Marcel Hörning, Torsten Bullmann, Tatsuo Shibata

**Affiliations:** ^1^Institute of Biomaterials and Biomolecular Systems, University of Stuttgart, Stuttgart, Germany; ^2^Laboratory for Physical Biology, RIKEN Center for Biosystems Dynamics Research, Kobe, Japan; ^3^Carl-Ludwig-Institute for Physiology, University of Leipzig, Leipzig, Germany

**Keywords:** dissipative structure, biochemical oscillation, small systems, signal transduction, chemotaxis, PIP3, membrane curvature, macropinocytosis

## Abstract

PIP3 dynamics observed in membranes are responsible for the protruding edge formation in cancer and amoeboid cells. The mechanisms that maintain those PIP3 domains in three-dimensional space remain elusive, due to limitations in observation and analysis techniques. Recently, a strong relation between the cell geometry, the spatial confinement of the membrane, and the excitable signal transduction system has been revealed by Hörning and Shibata (2019) using a novel 3D spatiotemporal analysis methodology that enables the study of membrane signaling on the entire membrane (Hörning and Shibata, 2019). Here, using 3D spatial fluctuation and phase map analysis on actin polymerization inhibited *Dictyostelium* cells, we reveal a spatial asymmetry of PIP3 signaling on the membrane that is mediated by the contact perimeter of the plasma membrane — the spatial boundary around the cell-substrate adhered area on the plasma membrane. We show that the contact perimeter guides PIP3 waves and acts as a pinning site of PIP3 phase singularities, that is, the center point of spiral waves. The contact perimeter serves as a diffusion influencing boundary that is regulated by a cell size- and shape-dependent curvature. Our findings suggest an underlying mechanism that explains how local curvature can favor actin polymerization when PIP3 domains get pinned at the curved protrusive membrane edges in amoeboid cells.

## 1. Introduction

Signal transduction systems exhibit a variety of self-organized pattern formations to control pivotal biological roles. Prominent examples are the oscillations of the Min proteins in *Escherichia coli* (Loose et al., 2011), orientation of cell polarity through regulation of the Cdc42 GTPase (Rho) cycle in yeast (Slaughter et al., 2009), the mechanochemical control of the asymmetric cell division by PAR proteins in *Caenorhabditis elegans* (Goldstein and Macara, 2007; Goehring and Grill, 2013), the directed migration of chemotactic eukaryotic cells (Devreotes et al., 2017), and the organization of macropinocytic cups (Buczynski et al., 1997; Hoeller et al., 2013; Veltman et al., 2016). Whereas, these dynamics maintain the natural life cycle of cells, there are also undesired miss-functions in biological systems in which signal transduction plays a crucial role. The most prominent examples are the cardiovascular system and cancerous tumors that can lead to life-threatening conditions. In cancer cells, signaling pathways are often altered in a way that results in uncontrolled growth and an increased capability to invade surrounding tissue (Bianco et al., 2006). Therefore, the understanding of the key signal transduction pathways that regulate the production of lipids and their spatiotemporal pattern evolution on the membrane are of crucial importance.

PI3 kinase is a critical regulator of cell survival and proliferation that controls the production of the membrane lipid PIP3 (Whitman et al., 1988), while PTEN, a lipid phosphatase, limits the increase in PIP3 (Sansal and Sellers, 2004). It is still under debate, whether PIP3 pathway play an indispensable role in gradient sensing under shallow chemoattractant gradients (Bosgraaf et al., 2008), or is independently regulated by PI3 kinase and thus pseudopod generation is controlled independently of chemotactic signaling (Andrew and Insall, 2007). However, PIP3 is also proposed to be linked to the formation of macropinocytic cups (Veltman et al., 2016), and therefore is involved in membrane remodeling. The spatiotemporal distribution of PIP3 has been studied extensively in migrating (Sasaki et al., 2007; Xiong et al., 2010) and actin polymerization-inhibited *Dictyostelium* cells (Arai et al., 2010; Shibata et al., 2012; Taniguchi et al., 2013; Nishikawa et al., 2014). The latter offers the advantage of studying the microscopic PIP3 domain dynamics under various perturbations without considering the spatiotemporal displacement and deformation of cells. The chemical inhibition of membrane deformation (change of local curvature) enables an easier identification of PIP3 dynamics that is critically influenced by PI3 kinase in the absence of the actin cytoskeleton (Janetopoulos et al., 2004; Xiong et al., 2010). However, most investigations are limited to a single focal plane and thus capture only a very limited fraction of the PIP3 dynamics (Sasaki et al., 2007; Arai et al., 2010; Xiong et al., 2010; Taniguchi et al., 2013; Nishikawa et al., 2014; Yamazaki et al., 2020), and therefore cannot provide comprehensive links to the influence of membrane curvature that may critically regulate PI3 kinase.

Recently, the PIP3 dynamics on entire cell membrane of *Dictyostelium* cells have been observed and analyzed using a novel automated computational methodology (Hörning and Shibata, 2019). The entire three-dimensional (3D) plasma membrane was localized and the corresponding PIP3 dynamics extracted using Delaunay triangulation and spherical harmonic analysis. It has been found that the dynamics of PIP3-enriched domains are influenced by subtle differences in cell shape (i.e., size and adhesion-mediated membrane distortion). Further, the velocity of the traveling wave depends on the refractory period, that is the time until the membrane becomes again excitable. In spatially extended systems, the refractory properties affect the behavior of traveling domain. In the case of *Dicyostelium* cell, the smaller the size of the cell, the slower the domain speed. This is because the cell membrane is a closed system that leads to the interaction of the domain front and rear. For very small cells the domain movement is suppressed and only localized transient spots can be observed. These self-regulatory effects of domain dynamics, which follow basic principles seen in other excitable media such as cardiac tissue (Campanari et al., 2017; Li et al., 2020) and Belousov-Zhabotinsky reactive medium (Courtemanche et al., 1993; Suematsu et al., 2011), have been confirmed using independent experiments of spatially constrained *Dictyostelium* cells that were embedded in narrow grooves of micro-chambers. The spatial constraint led to dominant domain circulations along the horizontal direction (parallel to the grooves) in contrast to the circulating waves along the periphery of cells that are adhered to flat substrates (Hörning and Shibata, 2019).

In this study, we took advantage of the high 3D spatial resolution that our previously introduced signal mapping routines offers (Hörning and Shibata, 2019). We investigated the local fluctuation of PIP3 signaling on the entire plasma membrane of the same *Dictyostelium* cells using detrended fluctuation analysis (DFA). We payed special attention to the edge of the ventral membrane, as it exhibits the largest membrane curvature on the actin-inhibited plasma membranes. We call this edge the contact perimeter, as it defines the border of the membrane in contact to the substrate and the membrane freely exposed to the culture medium. DFA allows us to quantify the underlying noise and signaling regime by determining the parameter α in the range of α = [0, 2] (Molz et al., 1997). The time series with value of α smaller than unity is a noise dominant process that has no underlying detectable signal. In contrast, when α is larger than unity, the time series is considered to contain an underlying signal (see example signals, Supplementary Figure 1). Those processes have been useful for interpreting various physiological or behavioral data (Delignières, 2015), such as heart-beat variability (Wallot et al., 2013), brain activity (Montez et al., 2009) and sensorimotor processes (Gilden et al., 1995). We mapped α over the entire surface of single cells using the local time series of PIP3 signaling measured before using a fluorescent indicator (Hörning and Shibata, 2019). We identified two different modes of the PIP3 domain dynamics in the observed cells, that are seemingly influenced by the contact perimeter. (1) Periodic PIP3 lipid-signaling (α>1) only on the upper membrane with noise dominant signaling (α~0.5) on the ventral membrane, and (2) periodic PIP3 lipid-signaling activity (α>1) that are anchored at the contact perimeter. The latter is observed in particular in larger cells that have a larger ventral membrane. We suggest that the contact perimeter serves as an anchor that can pin and guide propagating PIP3 domains, which means that the contact perimeter acts as an interactive boundary. The statistical analysis of the membrane shape and PIP3 domain dynamics showed that the strength of interaction between the domain and the contact perimeter is positively related to the degree of local membrane curvature at the contact perimeter, which might influence the local diffusivity between ventral and non-adherent membrane. That means a larger membrane curvature increases the effect of interaction between the PIP3 domain and the contact perimeter. We visualized those dynamics using high resolution phase analysis without spatial filtering.

## 2. Materials and Methods

*Dictyostelium discoideum* cells were used to observe spatiotemporal dynamics of PH_*Akt*/*PKB*_-EGFP molecules as they either bind to PIP3 on the membrane or move throughout to the cytoplasm. For this study we used the previously introduced signal mapping routines and the same cells that we observed in Hörning and Shibata (2019), as follows.

### 2.1. Cell Preparation

The GFP-fused pleckstrin-homology domain of Akt/PKB (PHAkt/PKB) was expressed in wild-type AX-2 cells. Cells were cultured at 21°C in HL-5 medium and selected with 20 μg/mL G418 (Watts and Ashworth, 1970). Before observation, the cells were placed in glass bottom dishes (IWAKI, Japan) 27 mm in diameter and starved in development buffer (DB: 5 mM Na phosphate buffer, 2 mM MgSO4, and 0.2 mM CaCl2, pH 6.3) for 4.5 h, leading to chemotactic competency with respect to cAMP (Arai et al., 2010). The glass bottom dishes were used in the presence or the absence of polyethylenimine (PEI) at a concentration of 2.4 μg/mL in DB. The number of cells was maintained at less than 5±10^5^ cells to sufficiently separate neighboring cells. After starvation the extracellular fluid was exchanged and the cells were incubated for an additional 20 min incubated before observation with 1 *m*L DB supplemented with 20 μM latrunculin A (L5163-100UG, Sigma), which inhibits actin polymerization, and 4*m*M caffeine, that inhibits the secretion of cAMP (Brenner and Thoms, 1984). A total of 79 cells were observed on glass and 98 cells on PEI-coated glass. The latter increases the adhesion strength of cells and leads therefore to a larger fraction of the plasma membrane adhering to the substrate (ventral membrane) (Hörning and Shibata, 2019).

### 2.2. Cell Observation

Fluorescence images were obtained using an inverted microscope (IX81-ZDC2; Olympus) equipped with a motorized piezo stage and a spinning disc confocal unit (CSU-X1-A1; Yokogawa) through a 60x oil immersion objective lens (N.A. 1.35; UPLSAPO 60XO). PH_Akt/PKB_-EGFP was excited by 488-nm laser diode (50 mW). The images were passed through an emission filter (YOKO 520/35, Yokogawa) and captured simultaneously by a water-cooled EMCCD camera (Evolve-, Photometrics). Time-lapse movies were acquired at 10-s intervals at a spatial resolution of dx = dy = 0.2666 μm and dz = 0.5 μm using z-streaming (MetaMorph 7.7.5), which enables recording the entire focal volume in about 3s.

### 2.3. 3D Reconstruction of Membrane Topology and Signals

The recorded spatiotemporal fluorescence signaling of cells was reconstructed considering the entire four-dimensional data set (*x*, *y*, *z* and *t*). The cell surface was represented by a triangular mesh that was generated using a Delaunay triangulation routine in MATLAB (Mathworks) (Persson and Strang, 2004), and transformed to a volume filling three-dimensional tetrahedral mesh by connecting the surface triangles to the center coordinate of the cell. The sections were evaluated by the time-averaged, radial intensity profile of all pixels within one grid element. The maximum intensity of the time-averaged standard deviation determined the membrane position. Thereafter, the position of nodes is determined, so that the average node area was approximately 0.3 μ*m*^2^. Finally, the grid nodes were smoothed among neighboring nodes using a triangular low-gaussian kernel. The PIP3 domain dynamics were quantified by spherical harmonic expansion to the 3rd order (octupole moment), which smoothened the intensity distribution for peak detection of the domains. The domain velocity, *v* is calculated from the temporal peak displacement on the membrane by approximating the peak-to-peak distance as the shortest distance along a straight line on the membrane. A more detailed methodological description can be found in Hörning and Shibata (2019). The generated data tables used for this study can be found in the Supplementary Material.

### 2.4. Mollweide Projection

The two-dimensional homolographic equal-area Mollweide projection is used to visualize membrane signaling in 2D. The transformation from spherical coordinates (ϕ, θ) to cartesian coordinates (*x, y*) is performed through the equations


(1) $$\begin{array}{r} x=\frac{2\sqrt{2}}{\pi }\theta \cos\gamma \end{array}$$

(2) $$\begin{array}{r} y=\sqrt{2}\sin\gamma \text{,} \end{array}$$

with the auxiliary angle γ given as


(3) $$\begin{array}{r} 2\gamma +\sin2\gamma =\pi \sin\phi \text{,} \end{array}$$

which can be solved iteratively by the Newton-Raphson method (Snyder, 1987; Hörning and Shibata, 2019).

### 2.5. Detrended Fluctuation Analysis

The statistical self-affinity of the extracted PIP3 signaling on the membrane of *Dictyostelium* cells was determined by Detrended Fluctuation Analysis (DFA) of first order (Peng et al., 1994). A previously introduced scheme in Matlab was used to determine the scaling exponent, α from the time series (Habib, 2017; Habib et al., 2017). The cumulated sum *S*(*t*_*i*_) of the discrete signal *s*(*t*_*i*_) is computed as


(4) $$\begin{array}{r} S_{i}=\overset{N}{\underset{i=1}{\sum }}s\left(t_{i}\right)-ŝ\text{,} \end{array}$$

where ŝ denotes the mean of *s*(*t*_*i*_). The variance *F*^2^(τ) is determined within the time window *T*_max_ = 180 s by obtaining the best linear fit *P* of the cumulated sum *S*, as


(5) $$\begin{array}{r} F^{2}\left(\tau \right)=\frac{1}{\tau }\overset{\tau }{\underset{i=1}{\sum }}{\left(S_{i}-P_{i}\right)}^{2}\text{.} \end{array}$$

Plotting the τ against *F*^2^(τ) on a log-log graph indicates α as the linear slope, since *F*(τ)~τ^α^. The extension of this framework for higher orders (i.e., DFAn) is explained elsewhere (Habib, 2017; Habib et al., 2017). Examples of different numerically generated and analyzed time series are shown in Supplementary Figure 1.

### 2.6. Phase Analysis

Phase information was extracted from the raw signals using the WAVOS toolkit for wavelet analysis and visualization of oscillatory systems in Matlab (Harang et al., 2012). The complex Morlet wave function


(6) $$\begin{array}{r} \psi \left(t\right)=\frac{1}{\sqrt{\omega }}\exp\left(i\omega t\right)\exp\left(-\frac{t^{2}}{2}\right) \end{array}$$

is applied to the PIP3 fluorescence intensity data at each membrane node using ω = 2π in the range of *t*_min_ = 60 s to *t*_max_ = 330 s. The dominant frequency in the power spectra was determined as the dominant frequency mode. The phase was calculated from the complex wavelet coefficients of the dominant frequency mode. Finally, all extracted phases were reconstructed and mapped on the membrane mesh.

## 3. Results

PIP3 domains self-regulate their dynamics on the spatially confined plasma membrane of actin polymerization-inhibited *Dicyostelium* cells. Those dynamics are determined by the geometrical shape of the membrane and the underlying excitability of the signal transduction system (Hörning and Shibata, 2019). While PIP3 domain dynamics were revealed using spherical harmonics analysis on the entire plasma membrane, the role of local signaling differences on the plasma membrane in influencing domain dynamics remains to be understood.

### 3.1. 3D Quantification of Cell Shape and Signaling

**Figure 1:**
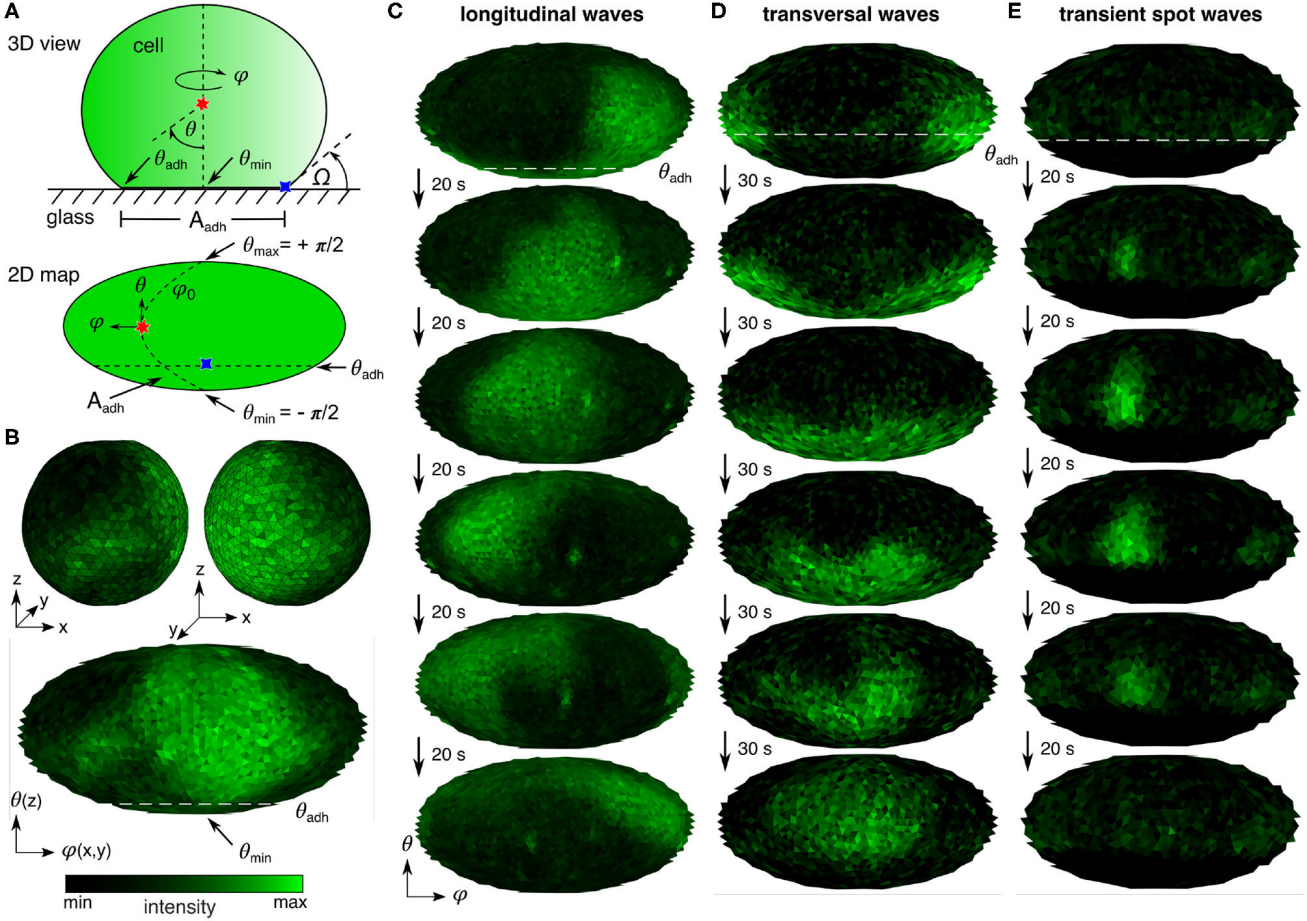
PIP3 domain dynamics on *Dictyostelium* cells. **(A)** Scheme of actin polymerization-inhibited cell attached to glass surface: 3D view and 2D map. Indicated are spherical coordinates, the periodic azimuthal angle ϕ (horizontal) and polar angle θ (vertical), the positions of θ at the center of ventral membrane θ_min_ = −π/2 and the contact perimeter θ_adh_, the contact angle Ω, and the area of the ventral membran *A*_adh_. **(B)** 3D visualization of entire membrane PIP3 signaling. The upper two panels show the front and back side of a cell. The lower panel shows the corresponding 2D map (Mollweide projection, see Materials and Methods) of the entire membrane signaling. **(C,D)** Examples of the basic PIP3 domain dynamics. θ_adh_ is indicated at the first map by the white dashed line. **(C)** Horizontal waves that propagate parallel to the contact perimeter. **(D)** Vertical waves that periodically cross the contact perimeter. **(E)** Transient spot waves that appear and disappear repeatedly above the contact perimeter.

In this study, actin polymerization-inhibited *Dicyostelium* cells were observed and analyzed by taking advantage of the simple and symmetric cell shape. For that cells were treated with 10 mM latrunculin A and 4 mM caffeine to suppress cell motility, formation of membrane protrusions, and cell-cell interactions (Arai et al., 2010). Thus, the cell membrane can be described and mapped using a spherical coordinate system with the polar angle θ and the periodic azimuthal angle ϕ (Figure 1A). Further, the cell shape was described by the contact angle Ω and the area of the ventral membrane, i.e., the area of the membrane that adheres to the substrate, *A*_adh_.

Due to the simple symmetric shape of the cells, it is also possible to project the three dimensional PIP3 signal activity onto a two dimensional map, as shown in Figure 1B. The upper two panels show the front and back side of a cell with irregular complex shaped PIP3 domains, and the lower panel shows the corresponding 2D map (Mollweide projection; see Materials and Methods) of the entire membrane signaling. For that a customized mapping routine was applied, as introduced in detail before by Hörning and Shibata (2019). Briefly, the temporal fluorescence variation of the signaling at each local position was used to specify the membrane position, from which a three-dimensional triangular map was constructed using a Delaunay triangulation routine (Persson and Strang, 2004) with a constant mesh size of approximately 0.3 μ*m*^2^. Depending on the cell size, this approach leads to membrane grids with 620–6,632 nodes, i.e., triangles. Additionally, part of the cells were cultured on polyethylenimine (PEI) coated glass bottom dishes to increase the adhesiveness of cells to the glass substrate and therefore having a wider range of different cell shapes. The ventral membrane area increased from *A*_Adh, glass_ = 35.6 ± 5.6 *μ*m^2^ to *A*_Adh, PEI_ = 51.9 ± 4.2 *μ*m^2^ (mean ± SE). Between the two different substrates no influence on the PIP3 signaling was observed.

Using this methodological approach the three basic PIP3 domain dynamics could be observed. The horizontal waves that propagate parallel to the contact perimeter (Figure 1C), vertical waves that periodically cross the contact perimeter (Figure 1D), and transient spot waves that are small domains that appear and disappear repeatedly at seemingly random positions at the membrane above the contact perimeter (Figure 1E). The corresponding movies can be found in the Supplementary Material.

### 3.2. Contact Perimeter Influences Domain Signaling

**Figure 2:**
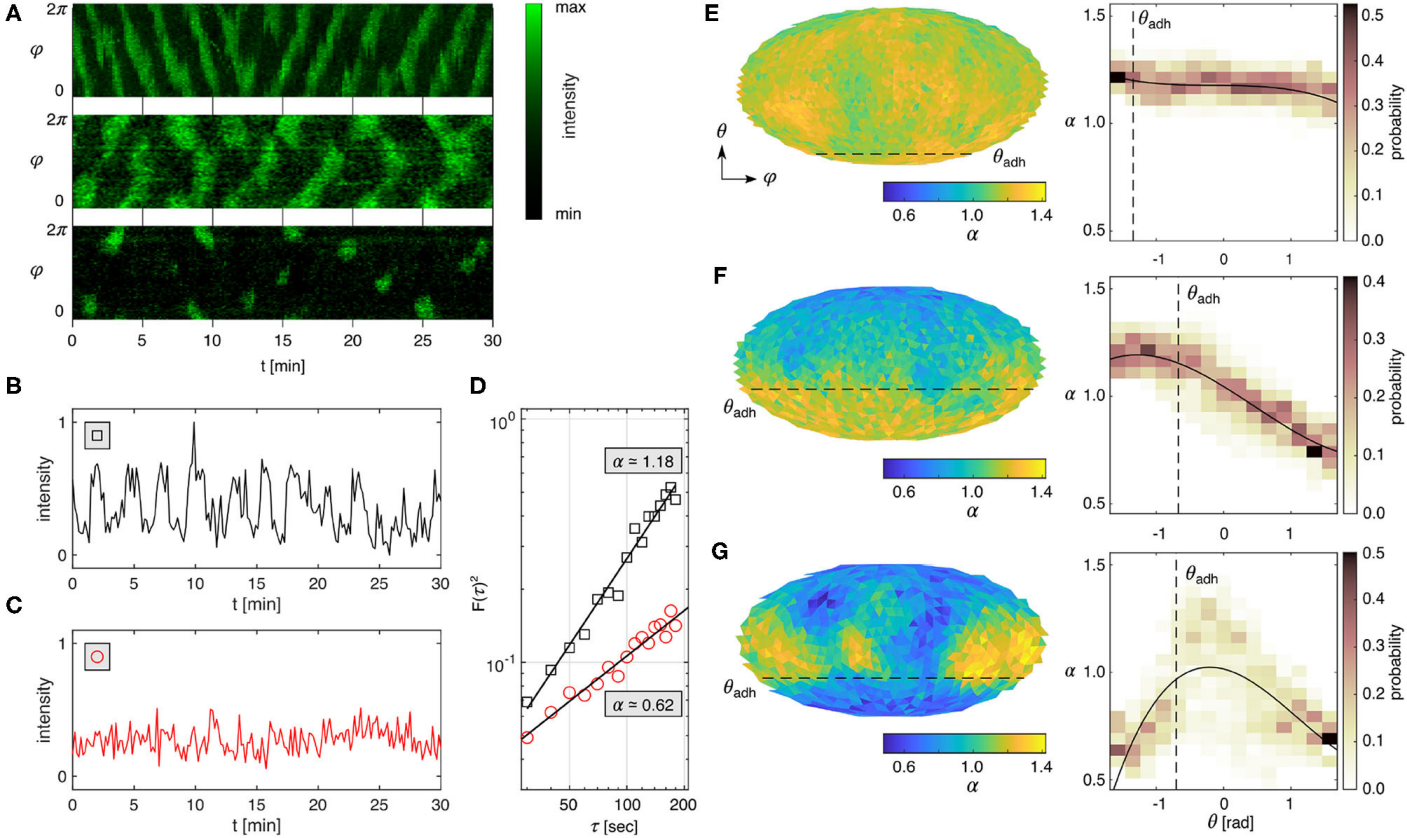
Spatial fluctuation analysis of PIP3 *Dictyostelium* membrane signals. **(A)** PIP3 dynamics recorded on the membrane periphery of *Dictyostelium* cells. Horizontal propagating waves (*top panel*), vertical propagating waves (*middle panel*), transient spot waves (*bottom panel*). Periodic signal **(B)** and noisy signal **(C)** taken at a membrane site that adheres to the substrate that correspond to the examples shown in **(A)** top and bottom panels, respectively. **(D)** Detrended fluctuation analysis of the signals shown in **(B,C)** showing noisy signal (α ≃ 1.18) and noise without detectable signal (α ≃ 0.62). **(E–G)** Mollweide maps of α for the three different prototypical PIP3 domain dynamics shown in **(A)**. The right panels show the respective histograms along the horizontal direction (θ) and the weighted polynomial fit (black solid line). The black dashed lines indicate the contact perimeter (θ_*adh*_) of the *Dictyostelium* membrane.

Figure 2A shows kymographs of the three PIP3 domain dynamics observed on *Dictyostelium* membranes: horizontal waves, vertical waves, and transient spot waves. While those wave patterns have been analyzed by tracking only the domain center using spherical harmonic analysis (Hörning and Shibata, 2019), the raw PIP3 signaling reveals irregular complex shaped domains (Figures 1B–E). Taking advantage of the high spatial resolution of the triangular mesh, it is possible to analyse the PIP3 signal at each triangle individually. Figures 2B,C show two typical periodic and non-periodic time series observed at single locations on different cells. Those signals can be independently analyzed by DFA. The resulting slopes enable the quantification of signal- and noise-dominant regimes in the range of α = [0, 2], where α = 0.5 corresponds to white noise and α = 2 to a signal without noise (see also Supplementary Figure 1). PIP3 signaling on *Dictyostelium* membranes was observed in the range of α ≃ [0.5, 1.5] similar to the shown periodic (α~1.2) and non-periodic (α~0.6) examples in Figures 2B–D.

**Figure 3:**
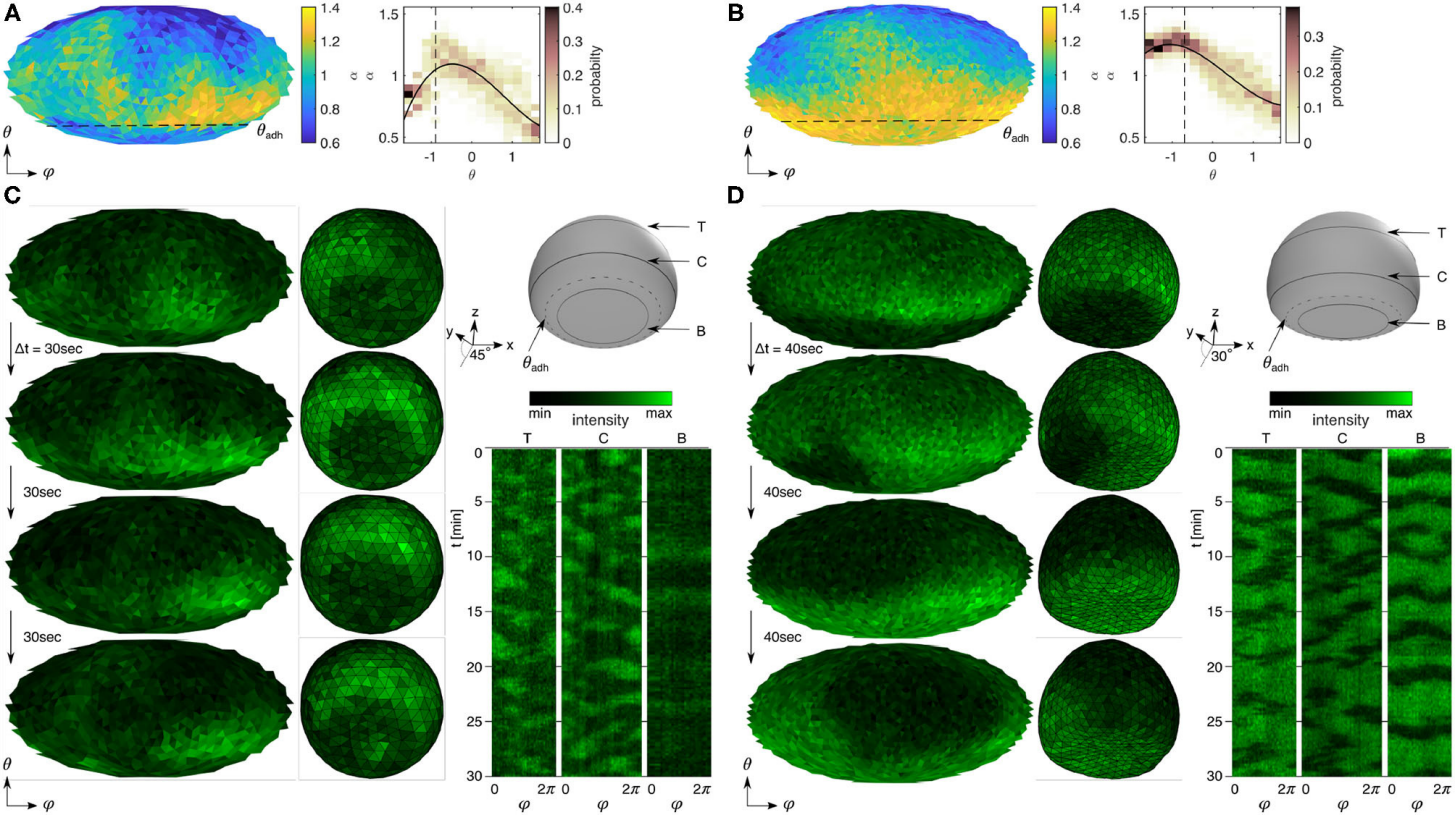
Influence of the contact perimeter on PIP3-enriched domain signaling. **(A,B)** Detrended fluctuation analysis of the cells that show noisy and oscillatory signaling on the ventral membrane sites (below dashed lines). The left panels show the Mollweide maps of α and the right panels show the respective histograms along the horizontal direction (θ) with the weighted polynomial fit (black solid line). See respective fits with confidence intervals in Supplementary Figure 2. **(C,D)** Spatiotemporal dynamics of PIP3-enriched domains shown in **(A,B)** indicating the contact perimeter (θ_adh_) as obstacle **(C)** and pinning site **(D)** of spiraling domains. The left panels are four snapshots—2D map and 3D view—of raw PIP3 signaling. The 3D view shows the ventral membrane from the bottom with a view angle of 45° and 30°. Three spatiotemporal kymographs of a bottom section [B], a center section [C] and a top section [T], as illustrated in the 3D cell scheme above. The dashed section illustrates the contact perimeter (θ_adh_). The sizes of ventral and entire membrane are **(C)** $A_{\text{adh}}=30\;\mu {\text{m}}^{2}$ and $A_{\text{mem}}=257\;\mu {\text{m}}^{2}$, and **(D)** $A_{\text{adh}}=105\;\mu {\text{m}}^{2}$ and $A_{\text{mem}}=564\mu {\text{m}}^{2}$.

Mapping α on the entire membrane (Figures 2E–G) reveals the underlying spatiotemporal pattern formations (Figure 2A). The yellow (α>1) and blue (α < 1) colors distinguish signal- and noise-dominant spatial locations on the membrane, respectively. The dashed line marks the contact perimeter. The right panels show the probability distribution of α along the vertical cell-symmetry axis from θ = −π/2 (cell bottom) to θ = +π/2 (top of cell) with the contact perimeter marked as a dashed line. Generally, we observed that smaller cells and those with a small ventral membrane area show more uniform distibutions of constant α (Figure 2E). Larger cells with a larger ventral membrane showed dominant periodic activity on the ventral membrane (Figure 2F), and smaller cells showed dominant periodic (yellow) activity at the upper plasma membrane with noisy activity below the contact perimeter (Figure 2G). The latter can be observed in very sharp transitions of activity above and below the contact perimeter (Figure 3A), implying an active role of the contact perimeter in PIP3 domain activity. Contrarily, the cell shown in Figure 3B shows constant values of α below the contact perimeter, and a gradual decrease of α toward the top of the cells (θ = π/2).

Figures 3C,D show time-series snapshots of PIP3 signaling corresponding to the two representative α-distribution maps (Figures 3A,B). Figure 3C illustrates how the contact perimeter blocks the domain motion toward the ventral membrane of the cell, which leads to domain guidance along the contact perimeter. The difference of domain activity gets more clear when comparing the kymographs above and below the contact perimeter (right panel). The latter shows no domain motion. However, in larger cells (Figure 3D), we observed that the domains can pass the contact perimeter and lead to domain motion on the entire membrane.

### 3.3. Correlation Between Membrane Geometry and Signaling

**Figure 4:**
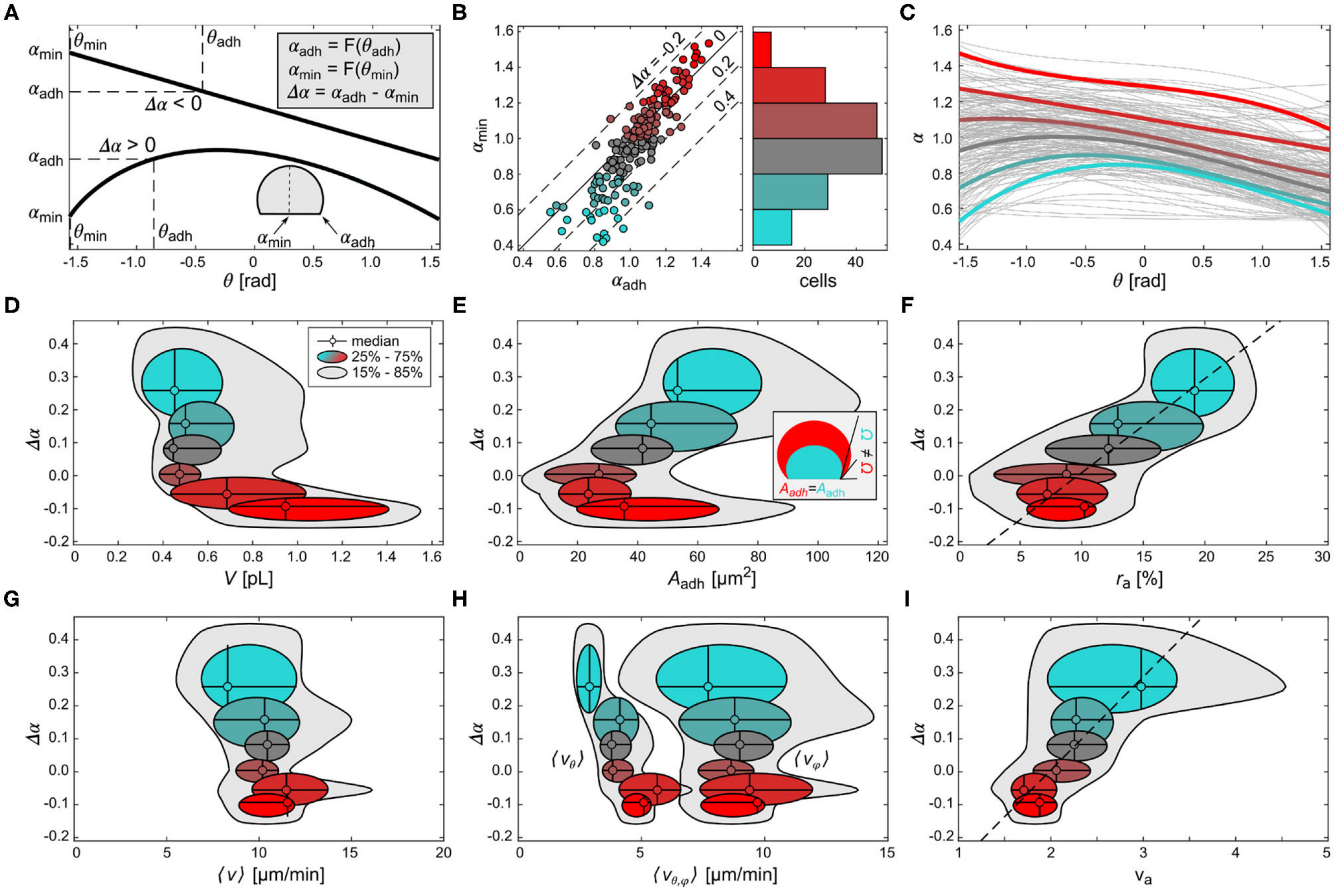
Comparison of fluctuation, geometry and domain dynamics between cells. **(A)** Scheme of two different weighted polynomial fits of α-histogram showing the minimal latitude (θ_min_) and contact perimeter (θ_adh_), and the respective levels of α (α_min_, α_adh_) that define the difference Δα. **(B)** The left panel shows the relation between α_min_ and α_adh_. The color scheme is applied as a function of α_min_. The right panel shows the number of cells that correspond to each α_min_ level, as a histogram. **(C)** The weighted polynomial fits of all cells (gray lines) and averaged representative fits of the binned α_min_ levels shown in the respective color. **(D–I)** The difference of α, Δα plotted as a function of the cell volume *V* in **(D)**, the ventral membrane area *A*_adh_ in **(E)**, the ratio between ventral and entire membrane area r_a_ = *A*_adh_/*A*_mem_ in **(F)**, the mean domain speed 〈v〉 in **(G)**, average vertical 〈v_θ_〉 and horizontal 〈v_ϕ_〉 wave speed in **(H)**, and the domain speed ratio v_a_ = v_φ_/v_θ_ **(I)**. The color scheme corresponds to the α_*min*_ levels shown in **(B)**. The dashed lines in **(F,I)** are the linear fits of the medians. The inset in **(E)** depicts the difference in membrane curvature, representatively shown by the contact angle Ω, for two cells of different volume but equal ventral area *A*_adh_.

In order to quantify the role of the contact perimeter in membrane signaling, a total of 178 cells were statistically analyzed with focus on the spatial distribution of α, as shown in Figures 3A,B (right panels). The weighted polynomial fits (black solid line) of the α distributions were parameterized by α_min_ and α_adh_, which define α at the polar angles θ_min_=−π/2 at the bottom of the cell and θ_adh_ at the spatial position of the contact perimeter, respectively (Figure 4A). Further, we introduce the scaling parameter Δα = α_adh_ − α_min_, which parameterizes the difference in signal activity between the bottom of the cell (θ_min_) and the contact perimeter (θ_adh_). A negative Δα is associated with a highly periodic or oscillatory signal at the ventral membrane (Figures 2F, 4A, upper black line), while a positive value indicates noise-dominant signaling at the ventral membrane (Figures 2G, 4A, lower black line). Figure 4B shows the relation between α_adh_ and α_min_, where the color scheme indicates cells of different α_min_ (see also Supplementary Figure 3). This color scheme provides an additional fingerprint of the membrane signaling independently from the shape of the weighted polynomial fits (Figure 4C). Cells that show Δα ≃ 0 are cells of comparable signaling at the center of ventral membrane and contact perimeter (α_min_ ~ α_adh_). Those cells are either dominated by periodic PIP3 signaling or only noise on the membrane. The latter are mainly very small cells that do not even exhibit transient spot dynamics, as shown in Figure 1E. Although this is a limitation of the parameter Δα, as those two types of cells are indistinguishable, they are only a very few cells whose PIP3 signaling is dominated by noise, and therefore statistically negligible (see Figure 4B, α_min_ ≃ α_adh_ ≃ 0.6; Figure 4C, α_min_ ≃ 0.6). Despite this limitation using Δα as scaling parameter, the major advantage is the normalization of the signaling strength relative to the contact perimeter, since there is a wide distribution of signal activity observed (Figure 4C).

Figures 4D–F show the relation between the scaling parameter Δα and the membrane geometry. We found that larger cells show an oscillatory signaling activity (Δα < 0) on the ventral membrane, while smaller cells show a broad distribution of noise dominant signaling activity in the range of Δα>0 (Figure 4D). Further, smaller cells (cyan color) show a linear correlation between Δα and the ventral membrane area *A*_adh_, i.e., larger *A*_adh_ led to larger Δα, and thus to noise-dominant signaling activity on the ventral membrane (Figure 4E). A similar relation was found for cells with negative Δα, where larger cells (red color) had larger *A*_adh_ compared to smaller cells *V*~0.5 pL. The presence of two branches implies a pitchfork-like bifurcation in Δα at Δα ≃ 0. This means that different cells with the same *A*_adh_ can take either a smaller value of Δα or larger value. This can be explained by the local membrane curvature at the contact perimeter. Cells of similar ventral membrane area, for example $A_{\text{adh}}≃60\;\mu m^{2}$, but different volumes (compare small cyan and large red data points) have different contact angles Ω, an indirect measure of the local membrane curvature at the contact perimeter. The inset of Figure 4E shows cells with different membrane curvature (see Discussion for more details). This finding suggests that the local membrane curvature at the contact perimeter regulates the diffusibility between the ventral and non-adherent part of the membrane. Considering the dimensionless adhesion area ratio of r_a_ = *A*_adh_/*A*_mem_, a linear relationship between r_a_ and Δα is found (dashed line, Figure 4F). Larger r_a_ lead to larger positive Δα. The corresponding bifurcation found for *A*_adh_ at Δα~0 corresponds to r_a_ ≃ 10%.

Similar to the geometry of the cells, we have compared the PIP3-enriched domain velocities (see Materials and Methods) to the signal fluctuations. Despite the differences in shape and size of the cell membranes, the average velocity was found to be about 〈v〉~10 μm/min suggesting comparable diffusion properties among all cells. Therefore, no dependence on membrane geometry was observed (Figure 4G). The mean horizontal velocity 〈v_φ_〉 shows a similar constant profile, and is the dominant fraction of 〈v〉 (Figure 4H). Contrarily, the mean vertical velocity 〈v_θ_〉 is by a factor of about two slower than 〈v_φ_〉, and linearly correlated with Δα, i.e., an increase of 〈v_φ_〉 is accompanied by an increase in cell size. Figure 4I shows Δα as a function of the mean velocity ratio 〈v_a_〉 = 〈v_φ_/v_θ_〉. A linear relationship is observed similar to the one in Figure 4F.

We conclude that the contact perimeter plays an important role for the pattern organization of the PIP3 signaling dynamics on the membrane. Using weighted polynomial fits, two relationships between the cell geometry (Figure 4F) and the PIP3 domain speed (Figure 4I) were found to be correlated linearly with the introduced contact perimeter dependent parameter Δα (Figure 4E). While it was shown before that the cell asymmetry induces directed domain propagation along the cell periphery (Hörning and Shibata, 2019), this result identifies the contact perimeter as an additional factor that controls the PIP3 signaling organization.

### 3.4. Guidance and Pinning of Waves at the Contact Perimeter

Thus, we summarize that not only the adhesion area ratio *r*_a_ but also the PIP3 domain velocity ratio v_a_ is linearly correlated to the scaling parameter Δα. From those two relationships (dashed lines, Figures 4F,I) we can derive the linear relation between r_a_ and v_a_ following the general linear equation v_a_ = *m* × r_a_ + *n*_0_, where *m* defines the slope and *n*_0_ the intersection with the y-axis at r_a_ = 0. Not surprisingly, we get the intersection at *n*_0_ ~ 1, since a perfect spherical cell (r_a_ = 0) will lead to a velocity ratio of unity (Supplementary Figure 4), as shown in numerical simulations before (Hörning and Shibata, 2019). In order to get a more intuitive view of the contact perimeter influence (Figures 2A,B), r_a_ is transformed to the contact angle Ω from the basic relationship


(7) $$\begin{array}{r} {\text{r}}_{\text{a}}=\frac{A_{\text{adh}}}{A_{\text{mem}}}=\frac{\pi R^{2}\sin^{2}\Omega }{4\pi R^{2}-\pi R^{2}\left(1-\cos\Omega \right)}\text{,} \end{array}$$

where *R* is the radius of the sphere. Equation 7 can be numerically solved as


(8) $$\begin{array}{r} \Omega =4\tan^{-1}\left(\sqrt{\frac{-2\sqrt{2{\text{r}}_{\text{a}}^{2}-3{\text{r}}_{\text{a}}+1}-3{\text{r}}_{\text{a}}+2}{{\text{r}}_{\text{a}}}}\right)\text{.} \end{array}$$

**Figure 5:**
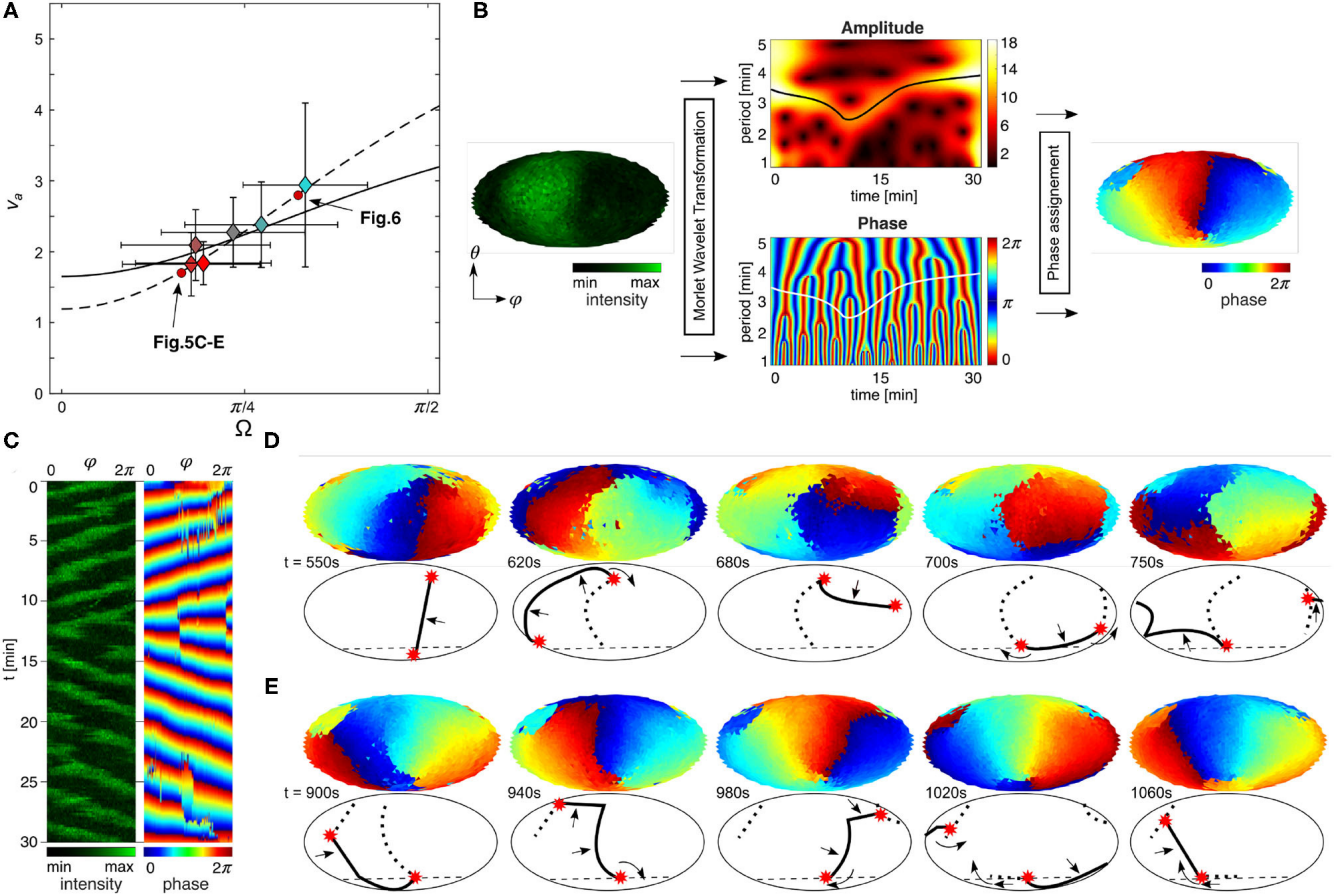
Curvature of contact perimeter acts as pinning site of PIP3 spirals. **(A)** Relation between Ω (contact angle) and v_a_ (speed ratio). Dashed line is the weighted fit calculated from the linear fits in Figures 4F,I. Solid line is the non-weighted fit of all cells without considering Δα (see also Supplementary Figure 4). Color coding corresponds to α_*min*_ (see Figure 4B). **(B)** Scheme of the phase extraction on entire membrane. Phase of oscillating PIP3 at every spatial point was extracted from fluorescence intensity (left map) by Morlet wavelet transformation [WAVOS] following the approach of Taniguchi et al. (2013). Amplitude and phase of the wavelet transform (center maps). The frequency component having the maximum amplitude at each time point was obtained by ridge extraction (black solid line), and the phase was calculated accordingly (white solid line). Spatially distributed phase is extracted (right map). **(C)** Kymographs of fluorescence intensity and phase maps. **(D,E)** Oscillating waves in nearly spherical cells (see Figures 1B, 2E). Phasemaps (top) and simplified schemes of the dynamics (bottom). **(D)** Change of domain front direction (solid line, 2π) from anticlockwise to clockwise wave direction by vertical phase line (dotted line) with one end pinned to the contact perimeter (dashed line). Phase singularities are denoted by red asterisks, and propagation direction with black arrows. **(E)** Spiral wave of PIP3 pinned to the contact perimeter (see lower red asterisk).

A good approximation for small contact angles (Ω ≃ 0) is $\Omega =-\sin^{-1}\left(2\sqrt{{\text{r}}_{\text{a}}}\right)$. Here, Ω is obtained purely based on geometric cell features, but does not account for minor membrane variations and possible constraints based on the mechanical properties of the membrane. Figure 5A shows the relation between Ω and 〈v_a_〉. The dashed line denotes the weighted polynomial fit derived from Figures 4F,I. At Ω = 0 we obtain v_a_ ≃ 1, as the theory predicts when considering isotropic diffusion on membranes. Contrarily the unweighted fit of the data (solid line), where cells with large *A*_adh_ (cyan) and large cells with comparable *A*_adh_ (red) are underrepresented (Figure 4B, left panel), leads to misleading statistics.

**Figure 6:**
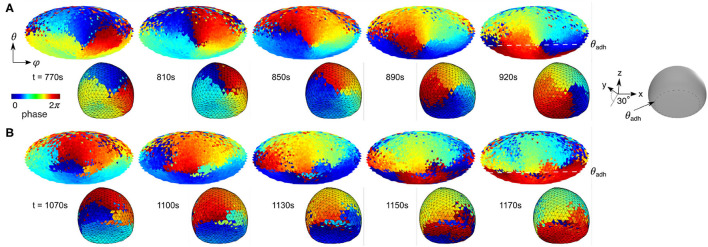
Contact perimeter guides PIP3 waves. **(A)** Contact perimeter serves as pinning site. **(B)** Contact perimeter guides PIP3 waves. Snapshots of phase maps are shown in 2D (top) and 3D (bottom). 3D maps are tilted 30° for showing the ventral membrane too, as shown by the gray 3D illustration where θ_adh_ is illustrated. The cell that is used to show this patterns is shown in Figures 3B,D, and its relation between contact angle Ω and speed ratio v_a_ marked by a red circle in Figure 5A.

To visualize the influence of the contact perimeter on the signaling dynamics, we utilized the Morlet wavelet transformation (Harang et al., 2012) at all spatially extracted positions on the membrane, as done before only for the ventral membrane of *Dictyostelium* cells (Taniguchi et al., 2013). Figure 5B shows a scheme of the phase extraction of the entire membrane from the raw data (left side) to the resulting phase map (right side). The phase is adjusted to the propagation front of the domain, so that the red color (= 2π) can be considered as wave front. Applied to the entire spatiotemporal raw data, it is also possible to generate kymographs and snapshots of the time-series, as illustrated in Figures 5C–E. The example illustrates the dynamics of vertical waves of an almost spherical cell (see Figure 2E). The advantage of this visualization method is that the PIP3 wave dynamics and phase singularities can be visualized clearly without application of spatial filters. Figure 5D shows the wave directional change from clockwise to anticlockwise rotation during the 10th and 15th min (Figure 5C) by rotation around a phase singularity (red asterisks), which we denote as a pinning site. The PIP3 wave propagates after the turn around the pinning site toward the center of ventral membrane along an elongated phase line. While the pinning site remains spatially anchored at the contact perimeter until *t* = 1, 020s (Figure 5E), there is no spatially anchored pinning site at the upper part of the membrane. That means that the PIP3 wave is guided along the contact perimeter, illustrated by the positional change of the phase singularity (red asterisks), because on the upper membrane is no strongly curved membrane as at the contact perimeter. Between *t* = 1, 020s and *t* = 1, 060s the phase singularity moves along the contact perimeter and a new pinning site is formed (last panel). Here, we observe two pinning sites, because of the topology constraints of spiral waves in excitable media (Davidsen et al., 2004). While one pinning site changes its position with time on the upper part of the membrane, the pining site on the contact perimeter does not change its position. This topology constraint together with the refractory behavior of the excitable signaling leads to an 〈v_a_〉 ~ 1.7, as marked in Figure 5A. Larger cells with large *A*_adh_ show an even more dominant influence of the contact perimeter on the PIP3 dynamics (Figure 6). Pinning sites do not only anchor to the contact perimeter (Figure 6A), but can also serve as wave guidance for the wave front (Figure 6B). In this case the PIP3 wave edge aligns with the contact perimeter and leads to even larger speed ratios by forcing the PIP3 wave to propagate parallel to the cell perimeter, in this example to almost 〈v_a_〉 ~ 3 (Figure 5A).

## 4. Discussion

Self-organization of signals in spatially extended systems are directly and indirectly influenced by the spatial topology. The regulation of reaction in 1D rings (Nishiyama, 1995), 2D rings (Li et al., 2020), and spherical surfaces (Maselko and Showalter, 1989) leads to self-organization of periodic waves, whose length scales are defined by the spatial confinement (Howard et al., 2011) and system size (Campanari et al., 2017). These examples highlight the strong correlation of wave patterning and system topology in confined excitable systems. Most of these systems are artificially formed and do not show large curvatures, such as membrane protrusions in migrating cells. However, recent studies on spatially confined *Dictyostelium* cells indicated the importance of membrane curvature, when observing that excitable dynamics are directly regulated by 3D geometry (i.e., size and shape) on the entire plasma membranes (Hörning and Shibata, 2019). Using the raw data observed on the entire plasma membrane and taking advantage of high-resolution signaling extraction methodology, we were able to statistically analyse and visually proof the importance of larger membrane curvature by using the contact angle Ω, as an indirect measure of the local membrane curvature at the contact perimeter. While the membrane curvature of actin-inhibited cells is not as large as in membrane protrusions, the curvature at the contact perimeter, hereon denoted as κ_max_, is sufficiently large to pin and guide the PIP3 waves. This leads to a curvature related diffusion change at the contact perimeter, i.e., smaller cells with larger κ_max_ lead to a boundary that restricts diffusion and larger cells with slightly lower κ_max_ lead to lower diffusion properties at the contact perimeter. Therefore, we speculate that the PIP3 waves that propagate to the tips of membrane protrusions (location of largest curvature) get pinned and can promote actin polymerization in *Dictyostelium* cells. Curvature-restricted diffusivity in *Dictyostelium* could be experimentally tested by the use of fluorescence recovery after photobleaching (FRAP). Bleaching the membrane close to the highly curved membrane, such as the edge of ventral membrane, should show a slower recovery than bleaching far from the curved membrane, as the diffusion is restricted by the contact perimeter. This effect should increase with an increase of the contact angle, which can be experimentally controlled by the amount of PEI.

A previous study on *Dictyostelium* cells that were confined between two planar surface reported that a PIP3 wave was formed on either side of the opposing cell surface with switching period of 2–5 min (Helenius et al., 2018). The switching time scale is comparable to the period of PIP3 observed in the present study (see Figures 2A,B, 5B). Such an experimental setup could be used to study the influence of the membrane curvature on the PIP3 domain dynamics in more detail by adjusting the distance between two planar surface.

In the present study, we did not observed the PIP3 domain formation that was confined only in the ventral membrane. This indicated that the entire membrane region (both ventral and non-adhesive membrane regions) have the excitable property. However, we expect that a confined PIP3 wave within either ventral or non-adhesive membrane regions can be formed if the curvature at the boundary between two regions is sufficiently high and due to the inhibition of multiple PIP3 domain formation through cytosol interaction (lateral inhibition) (Gerisch et al., 2012; Taniguchi et al., 2013).

In this study we showed an organizing role of the contact perimeter on the membrane pattern formation. To clarify the mechanism, it is necessary to study how the contact perimeter interacts with the membrane molecules. In particular, PIP3 is known to accumulate on the membrane in a mutually exclusive manner with PTEN. For this sharp transition between the two states, a mutual inhibitory mechanism between PIP3 and PTEN plays an important role (Arai et al., 2010; Gerisch et al., 2012; Matsuoka and Ueda, 2018). In addition to such direct interaction between these molecules, the membrane curvature might play some role on the sharp separation by changing the membrane localization of the regulatory molecules.

Following on that, another important aspect of PIP3 dynamics is the macropinocytosis (Buczynski et al., 1997; Hoeller et al., 2013; Veltman et al., 2016), which is a unique pathway of endocytosis to internalize large amounts of extracellular fluid, solutes and membrane (Lin et al., 2020). In non-mammalian cells, such as *Dictyostelium* cells, macropinocytosis requires PIP3 and the persistent activation of Ras Williams et al. (2019) to form patches in macropinosomes Ritter et al. (2021). While this pathway is also observed in diverse range of mammalian cells too, such as immune cells, endothelial and epithelial cells (Lin et al., 2020), they also play an essential role in cancer cells as reviewed in Ha et al. (2016), Recouvreux and Commisso (2017), Zhang and Commisso (2019), and Ritter et al. (2021). It has been identified that macropinocytosis is a mechanism by which cancer cells support their unique metabolic needs (Commisso et al., 2013), and has therefore increased the interest for anticancer therapies (Ritter et al., 2021; Song et al., 2021). The findings presented here open an interesting new aspect to macropinocytosis that suggests relevance to cancer cells too, as PIP3 domain dynamics are observed as self-organizing waves that are prone to be pinned and guided by strongly bended membranes, just as observed during the formation of macropinocytic cups (Veltman et al., 2016).

Another possible explanation can be given by considering molecular crowding (Liu et al., 2018) and induced changes in lipid composition and their asymmetry (Bigay and Antonny, 2012; McMahon and Boucrot, 2015) at the contact perimeter, which may even lead to local conformational switches (Aisenbrey et al., 2008) and nano-domain clustering, as observed for glycolipid GM3 and PIP2 in molecular simulations of complex lipid bilayers (Koldsø et al., 2014). The large headgroups in PIP3 confers an inverted conical shape to the lipids, thereby favoring the bending of the membrane into a positive curvature (Di Paolo and De Camilli, 2006; McMahon and Boucrot, 2015). As for actin-inhibited *Dictyostelium* cells, the plasma membrane is immobilized and larger curvature is observed at the contact perimeter only. While the membrane area ratio r_a_ can be comparable for cells of different volumes, κ_max_ depends on the cell volume and ventral membrane size. Thus, larger cells have smaller κ_max_. Therefore, in larger cells the local diffusivity of PIP3 is reduced at the contact perimeter (Figure 3B), while the contact perimeter of smaller cells with larger κ_max_ serves as a diffusion boundary (Figure 3A).

**Figure 7:**
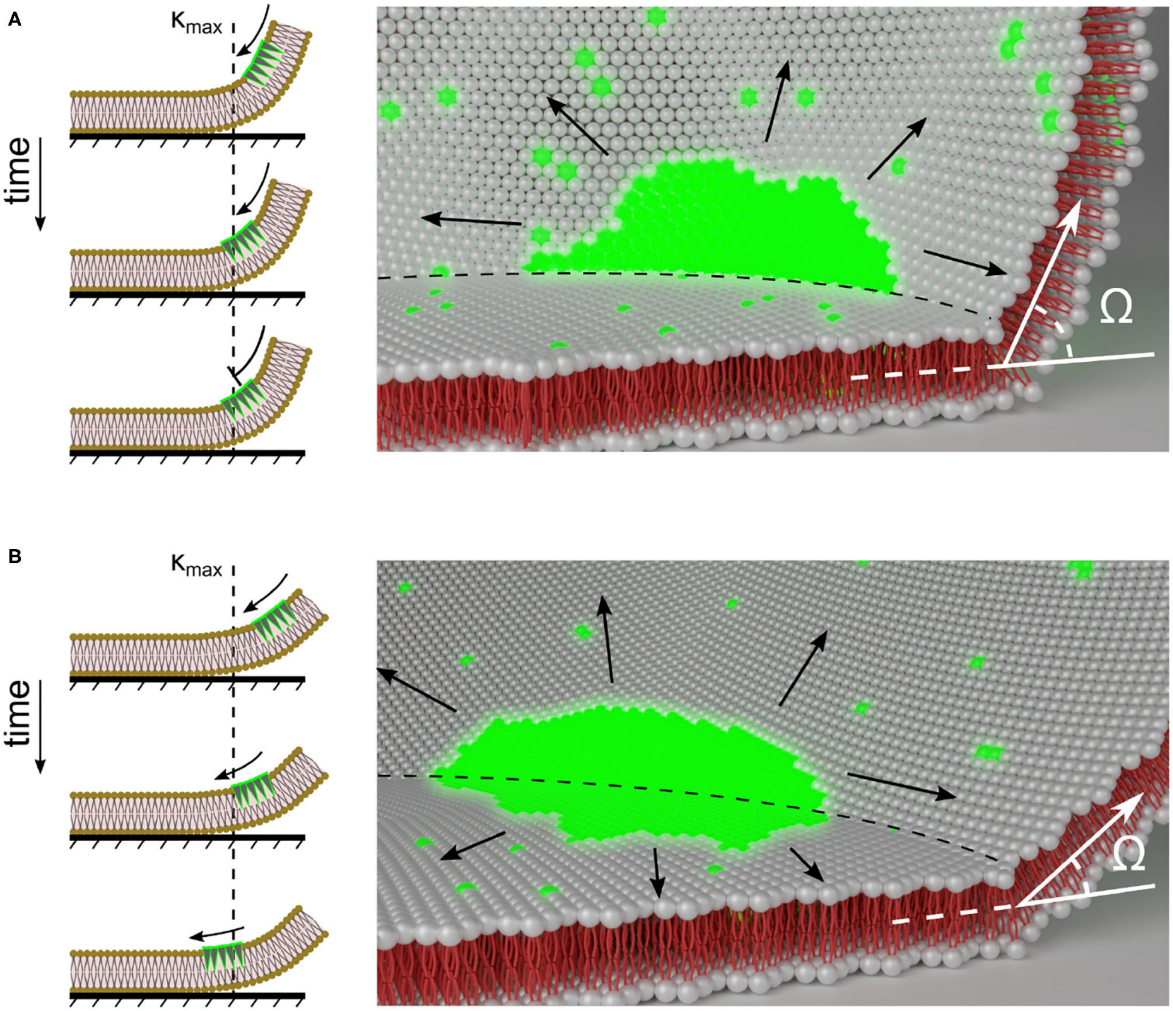
Scheme of membrane curvature regulated PIP3-domain signaling at the contact perimeter. PIP3-domain diffusion across the contact perimeter depends on the degree of local membrane curvature. **(A,B)** show illustrations of differently sized cells that have large and small membrane curvatures, κ at the contact perimeter, and the influence of the contact perimeter on the PIP3-domain (green) propagation. The left panels show the one dimensional view of PIP3-domain propagation. κ_max_ shows the position of the contact perimeter and the membrane location with highest degree of curvature. The black arrows indicate the direction of PIP3-domain propagation. The right panels show the two dimensional view. The black arrows indicate possible directions of PIP3-domain propagation that may also lead to anchored waves at the contact perimeter (dashed line). The contact angle Ω is representatively shown at the contact perimeter.

One possible explanation of these findings could be the influence of the large polar PIP3 headgroups that may interact and disturb the free diffusibility at the strongly curved contact perimeter and subsequently lead to molecular crowding (see scheme in Figure 7). This crowding may affect compartmentalization and other mechanisms in living cells that allow proteins to sense, stabilize or generate a high local membrane curvature (McMahon and Boucrot, 2015), such as PTEN, a cytosolic protein that associates with the plasma membrane. Further experimental investigations on precisely controlled curvatures of artificial cell membrane systems using microfabrication (Komiya et al., 2020) and high-precision 3D printing (Erben et al., 2020) may enhance our understanding of these findings.

## Data Availability Statement

The raw data supporting the conclusions of this article will be made available by the authors, without undue reservation. A basic version of the implemented code is available at MATLAB Central File Exchange (Hörning, 2021).

## Author Contributions

MH, TB, and TS conceptualized and designed research and wrote the manuscript. MH and TS performed data acquisition. MH implemented the analysis routines and analyzed the data. All authors contributed to the article and approved the submitted version.

## Funding

This study was supported by the core funding at RIKEN Center for Biosystems Dynamics Research (to TS) and the Foreign Postdoctoral Researcher program at RIKEN (to MH).

## Conflict of Interest

The authors declare that the research was conducted in the absence of any commercial or financial relationships that could be construed as a potential conflict of interest.

## Publisher's Note

All claims expressed in this article are solely those of the authors and do not necessarily represent those of their affiliated organizations, or those of the publisher, the editors and the reviewers. Any product that may be evaluated in this article, or claim that may be made by its manufacturer, is not guaranteed or endorsed by the publisher.
