# Supplementary material for: Local Membrane Curvature Pins and Guides Excitable Membrane Waves in Chemotactic and Macropinocytic Cells - Biomedical Insights From an Innovative Simple Model
Source: Front Cell Dev Biol. 2021 Sep 15;9:670943. doi: 10.3389/fcell.2021.670943 (PMC8479871; doi:10.3389/fcell.2021.670943)
Supplement: Supplementary file 4 [file Data_Sheet_1.pdf]

Supplementary Material

1 SUPPLEMENTARY TABLES AND FIGURES

1.1 Figures

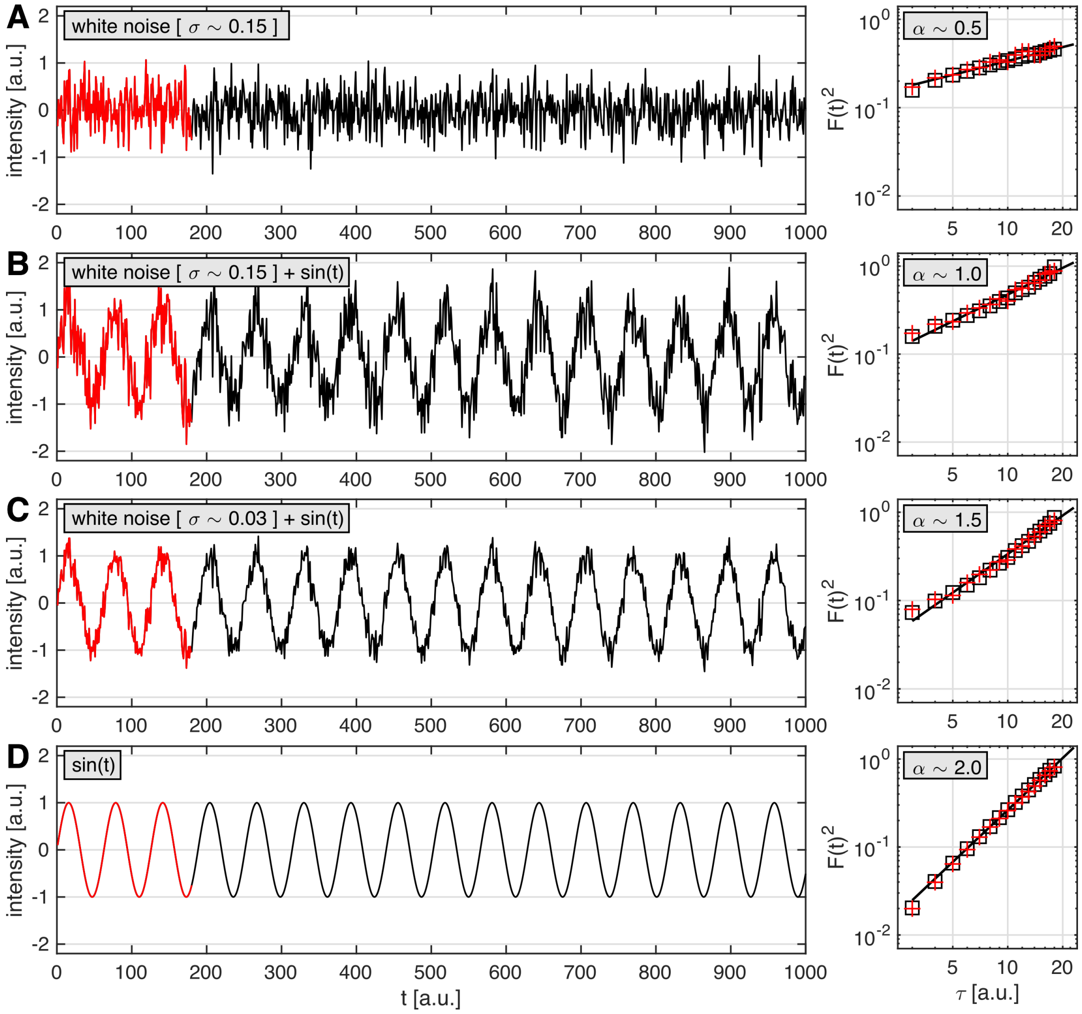

Figure S1. Fluctuation analysis of illustrative example signals. Four different signals (left panels) with respective detrended fluctuation analysis (right panels) are shown (**A**-**D**). The black time series' have a length of 1000 time points and the red series' are identically to the first 180 time points of the black series'. (**A**) Shown is white noise with a standard deviation of about $\sigma \simeq 0.15$ that corresponds to a slope parameter $\alpha \simeq 0.5$. (**B**) Shown is a sinus signal with white noise ($\sigma \simeq 0.15$) that corresponds to $\alpha \simeq 1.0$. (**C**) Shown is a sinus signal with weaker white noise ($\sigma \simeq 0.03$) that corresponds to $\alpha \simeq 1.5$. (**D**) Shown is a sinus signal without noise that corresponds to $\alpha \simeq 2.0$. Black and red data lead to comparable α, as illustrated by the slopes in black squares and red pluses in the right panels.

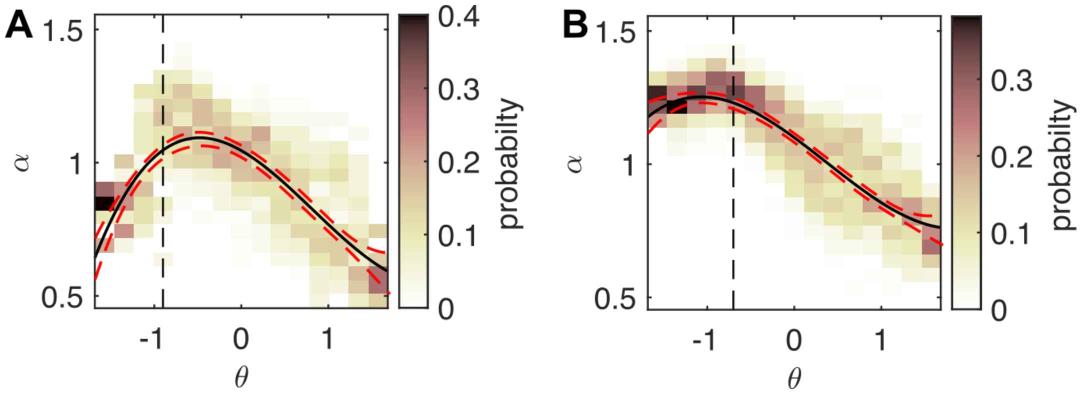

Figure S2. Detrended fluctuation analysis of the cells shown in Fig. 3. Show are the DFA histograms along the vertical direction (θ) with the weighted polynomial fit (black solid line) and the confidence intervals (red dashed lines).

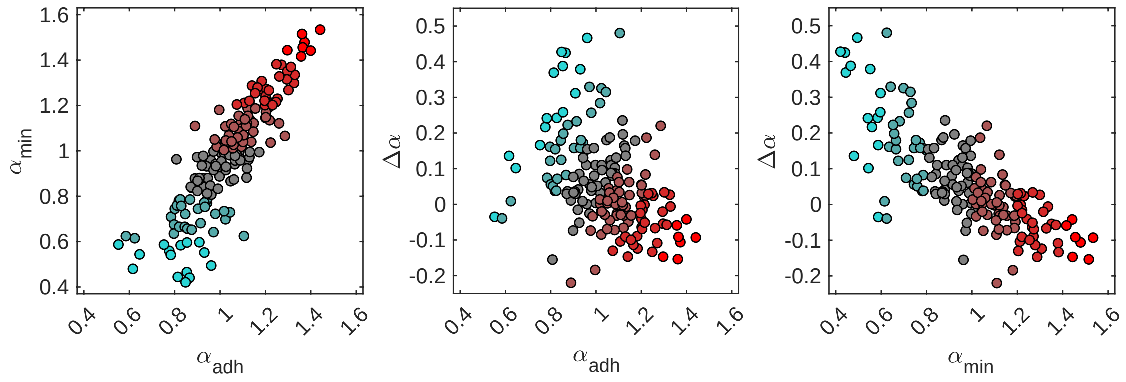

Figure S3. Shows an overview of the relationship between the from the DFA calculated parameters α_{min}, α_{adh} and $\Delta\alpha$.

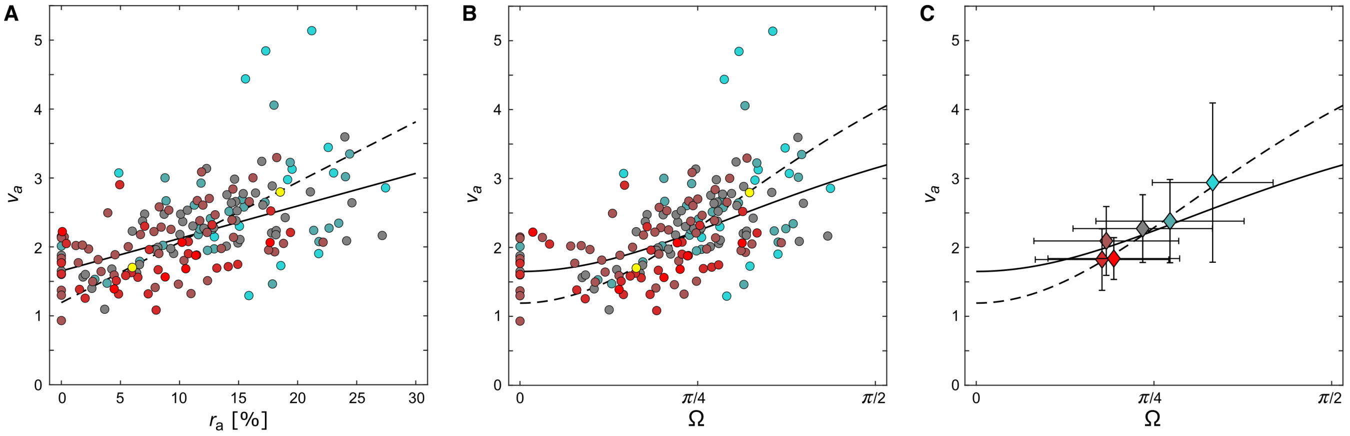

Figure S4. (**A**) Dashed line is the weighted fit calculated from the linear fits $r_a = A_{adh}/A_{mem}$ (Fig. 4F) vs. $v_a = v_\varphi/v_\theta$ (Fig. 4I). Solid line is the non-weighted fit of all cells without considering $\Delta\alpha$. Color coding corresponds to α_{min} (see Fig. 4B). (**B**) Transformation to membrane contact angle Ω (Eq. 8). Yellow points correspond to dynamics of cells shown in Fig. 5 and 6 (**C**) Mean and standard deviation of data plotted in (**B**).
